# Effect of *Cone of Pinus densiflora* on DNCB-Induced Allergic Contact Dermatitis-Like Skin Lesion in Balb/c Mice

**DOI:** 10.3390/nu13030839

**Published:** 2021-03-04

**Authors:** Boguen Kwon, Soo Yeon Hong, Eun-Young Kim, Jae-Hyun Kim, Minsun Kim, Jae Ho Park, Youngjoo Sohn, Hyuk-Sang Jung

**Affiliations:** 1 Department of Anatomy, College of Korean Medicine, Kyung Hee University, 26, Kyunghee dae-ro, Dongdaemun-gu, Seoul 02447, Korea; kbg6165@gmail.com (B.K.); ghdtndus121@naver.com (S.Y.H.); turns@hanmail.net (E.-Y.K.); jhk1@khu.ac.kr (J.-H.K.); alstjs8644@naver.com (M.K.); 2 Department of Pharmaceutical Science, Jungwon University, 85, Munmu-ro, Goesan-eup, Goesan-gun, Chungbuk 28024, Korea; parkjh@jwu.ac.kr

**Keywords:** *Cone of Pinus densiflora*, allergic contact dermatitis, HaCaT, HMC-1, MAPK, NF-κB, TARC, MCP-1

## Abstract

*Cone of Pinus densiflora* (CP), or Korean red pinecone, is a cluster of *Pinus densiflora* fruit. CP has also been verified in several studies to have anti-oxidation, anti-fungal, anti-bacterial, and anti-melanogenic effects. However, anti-inflammatory effects have not yet been confirmed in the inflammatory responses of pinecones to allergic contact dermatitis. The purpose of this study is to prove the anti-inflammatory effect of CP on allergic contact dermatitis (ACD) in vitro and in vivo. CP inhibited the expression of TSLP, TARC, MCP-1, TNF-α, and IL-6 in TNF-α/IFN-γ-stimulated HaCaT cells and MCP-1, GM-CSF, TNF-α, IL-6, and IL-8 in PMACI (phorbol-12-myristate-13-acetate plus A23187)-stimulated HMC-1 cells. CP inhibited the phosphorylation of mitogen-activated protein kinase (MAPKs), as well as the translocation of NF-κB on TNF-α/IFN-γ stimulated in HaCaT cells. In vivo, CP decreased major symptoms of ACD, levels of IL-6 in skin lesion, thickening of the epidermis and dermis, infiltration of eosinophils and mast cells, and the infiltration of CD4^+^ T cells and CD8^+^ T cells. This result suggests that CP represents a potential alternative medicine to ACD for diseases such as chronic skin inflammation.

## 1. Introduction

Allergic contact dermatitis (ACD) is a chronic inflammatory disease with urtication, xeroderma, and severe eczema lesions as its main symptoms. In severe cases, it causes psychological and emotional pain and sleep disturbance. ACD develops due to immune system abnormalities and the influence of environmental factors such as smoking, acarinosis, and air pollutants. This disease affects about 15 to 20% of infants and 1 to 3% of adults [1]. It was reported that about 80% of patients who develop allergic contact dermatitis in infancy maintain ACD through adulthood [2].

Keratinocytes are the cells that form the skin [3]. The keratinization of keratinocytes is precisely programmed and acts as a barrier to protect the skin [3]. However, if the skin barrier function is defective due to the abnormal differentiation of keratinocytes, the skin barrier function is lost due to the keratinization of keratinocytes, and the invasion of allergens initiates an inflammatory response from the keratinocytes [4,5]. The skin can cause several inflammatory skin diseases, such as allergic contact dermatitis, through the inflammatory reaction of keratinocytes [4]. Keratinocyte plays an important role in skin disease, in which HaCaT cells are mainly used for anti-inflammatory skin disease drug experiments [6].

Mast cells are immune cells that are a major factor in allergic and inflammatory responses [7]. Mast cells contain various allergens, cytokines, and chemokines that are released into the cells via the process of degranulation [8]. Mast cells are mainly present in the epidermis, immediately below the epidermis, or within the epidermis and are involved in inflammatory reactions in the skin [9]. Moreover, Mast cells are responsible for the main operative responses of innate and acquired immunity and in the defense against some bacteria, viruses, and parasites [10,11].

In ACD, TSLP (thymic stromal lymphopoietin) is a cytokine involved in the initiation, development and progression of atopic disease in both mice and humans [12]. It is produced by damaged epithelial cell and activates myeloid dendritic cells [13]. Activated dendritic cells promote the differentiation of naïve CD4+ cells into a Th2 phenotype cell. TSLP-activated dendritic cells produce Th2 attracting chemokine, such as TARC [14]. 

Thymus and activation-regulated chemokines (TARC and CCL17); the CCR4 ligands involved the movement of CCR4+ Th2 cells and monocyte chemoattractant protein-1 (MCP-1); monocytes, T cells, and basophil-related chemokines of C-C; and the beta family are all important chemokines that move inflammatory cells to the skin lesion [15,16]. They are often secreted by the inflammatory responses of keratinocytes and mast cells [4,17]. TARC is bound to CCR4 and induces the infiltration of Th2 lymphocytes in acute phase ACD. MCP-1 regulates the migration and infiltration of eosinophils, monocytes, and macrophages and is significantly increased in patients with psoriasis and other skin diseases [15,18]. These cytokines, such as TARC and MCP-1, trigger inflammation and play a role in maintaining inflammation [19,20]. When allergens infiltrate the skin, mast cells secrete IL-6 to induce the maturation of Th2 cells to promote inflammatory reactions, eosinophil infiltration, and hyperkeratosis of the epidermis [7,21]. GM-CSF promotes both the maturation and migration from epidermal Langerhans cells (LCs) and, subsequently, the migration of these LCs to draining lymph nodes to activate T lymphocytes [22]. Moreover, the skin’s GM-CSF levels are associated with variations in the severity of ACD [22]. The secretion of IL-8 promotes an inflammatory response by hampering the ability to inhibit T cell-mediated inflammation and brings neutrophils to the lesion site to intensify the inflammatory response [23]. Further, mast cells expand blood vessels and increase permeability, thereby constructing an environment in which mast cells or other inflammatory cells can easily penetrate the lesion site [24,25].

Specific cytokines of HaCaT cells have been demonstrated, in several studies, to activate the intracellular inflammatory response signaling pathways [26,27,28,29]. MAPKs, which is the most widely studied intracellular signaling cascade response in the inflammatory responses of HaCaT cells, generally consists of extracellular signal-regulated kinase (ERK), c-Jun N-terminal kinase (JNK), and P38 subgroups [30]. ERK performs an important role in the survival, proliferation, and differentiation of cells [30,31]. JNK facilitates cell death through apoptosis and cell differentiation [31]. P38 performs the most important role in the inflammatory reaction and is activated in various types of cell stress [30,31]. In the NF-κB/IκBα signaling pathway, which is well-known to be downstream of MAPK, NF-κB is generally combined with IκBα to form an inactive NF-κB complex [32]. However, IκBα, an NF-κB inhibitory protein, is phosphorylated upstream. The phosphorylation of IκBα causes poly-ubiquitination and degradation via the 26S proteasome, resulting in phosphorylation of NF-κB and translocation to the nucleus [33]. Nuclear translocated NF-κB is involved in the expression of numerous genes involved in the function and development of various immune system and inflammatory responses [32,33,34]. 

This study used a *Cone of Pinus densiflora* (CP), or a Korean red pine cone, which is a cluster of the *Pinus densiflora* fruit. Recent studies have shown the anti-fungal, anti-oxidant, anti-bacterial, anti-melanogenic, and anti-viral effects of pinecones [35,36,37,38,39,40]. In addition, studies on terpenes and tannins, the main constituents of pinecones, have also been actively conducted. Terpenes have shown to inhibit antioxidant effects in vitro, and tannins have also been reported to exert anti-inflammatory effects by inhibiting MAPKs and NF-κB pathways in a TNF-α-mediated cell model [41,42,43,44]. However, anti-inflammatory effects have not yet been confirmed in the inflammatory responses of pinecones to allergic contact dermatitis. The present study hypothesized that pinecones would have anti-inflammatory effects based on the results of research on the antioxidant effects of CP and the anti-inflammatory effects of the major components of CP. Therefore, this experiment was designed while predicting that pinecones would have an effect on keratinocyte hyper-keratinization and inflammatory responses due to the inflammatory responses of allergic contact dermatitis and the inflammatory responses of the mast cell.

To perform the experiment, cell experiments were conducted on HaCaT cells and HMC-1 cells using keratinocytes and mast cells, which play an important role in ACD. Animal experiments were performed using a mouse model of ACD-like skin lesions by treating Balb/c mice with 1-Chloro-2,4-dinitrobenzene (DNCB) to establish an ACD-like skin lesion model. Thus, this study demonstrated the effects of CP in TNF-α/IFN-γ-stimulated HaCaT cells, PMACI (phorbol-12-myristate-13-acetate plus A23187)-stimulated HMC-1 cells, and DNCB-induced ACD-like skin lesions in a Balb/c mouse model.

## 2. Materials and Methods

### 2.1. Reagents

HaCaT cells were purchased from Cell Line Service (CLS, Eppelheim, Germany). HMC-1 cells were purchased from American Type Culture Collection (ATCC, Manassas, VA, USA). Dulbecco’s Modified Eagle’s Medium (DMEM) and Dulbecco’s phosphate buffered saline (DPBS) were purchased from Welgene Biotech Co (Welgene bio Co, Gyeongsan, Korea). The antibiotics penicillin/streptomycin (P/S) and trypsin were obtained from Gibco (Carlsbad, CA, USA). Fetal bovine serum (FBS) was purchased from ATLAS Bio (Seoul, Korea). The 3-(4,5-dimethylthiazol-2-yl)-5-(3-carboxymethoxyphenyl)-2-(4-sulfophenyl)-2H-tetrazolium assay kit (MTS assay kit) was supplied by Promega (Madison, WI, USA). Anti-bodies of extracellular signal-regulated kinase (ERK), phosphorylation-ERK (p-ERK), P38, phosphorylation-P38 (p-P38), c-Jun N-terminal kinase (JNK), phosphorylation-JNK (p-JNK), nuclear factor kappa B subunit 1 (NF-κB), phosphorylation-NF-κB (p-NF-κB), NF-kappa-B inhibitor α (IκBα), and phosphorylation-IκBα (p-IκBα) were purchased from Cell signaling (Danvers, MA, USA). The protease inhibitor cocktail 1 (PI_1_, #P8340), phosphatase inhibitor cocktail 2 (PI_2_ #P5726), phosphatase inhibitor cocktail 3 (PI_3_, #P0044), and 1-Chloro-2,4-dinitrobenzene (DNCB, 237329) were purchased from Sigma-Aldrich (St. Louis, MO, USA). The NE-PER™ Nuclear and Cytoplasmic Extraction Reagent kit, bicinchoninic acid protein assay kit, and superscript Ⅱ reverse transcriptase were purchased from Thermo Fisher Scientific (Waltham, MA, USA). The nitrocellulose blotting membrane and ECL solution were purchased from General Electric Healthcare Life Sciences (GE Healthcare Life Sciences, Seoul, Korea). Interleukin-8 (IL-8), IL-1β, IL-6, IL-4, granulocyte-macrophage colony stimulating factor (GM-CSF), monocyte chemoattractant protein-1 (MCP-1), and tumor necrosis factor-alpha (TNF-α) were supplied by Becton, Dickinson bio (BD bio, NJ, USA). PCR primers was obtained from Genotech Corp (Daejeon, Korea). The Taq polymerase kit was supplied from Kapa Bio systems (Wilmington, MA, USA). Anti-bodies of CD4 (ab183685) and CD8 (ab209775) were purchased from Abcam (Cambridge, UK).

### 2.2. Preparation of the Cone of Pinus densiflora

The *Cone of Pinus densiflora*-ethyl acetate extract (CP) used in this study was collected and identified (voucher number: CLP09-27) at Chollipo Arboretum in Taean-gun, Chungcheongnam-do; the cones were provided by Professor Jaeho Park of Joongwon University and used as samples. CP was extracted by sonication for 3 days with 100% methanol. The extract was filtered (no. 3; Whatman, Maidstone, UK) and concentrated with a vacuum evaporator (N-1110S, EYELA, Shanghai, China). The methanol extract was then fractionated using petroleum ether and ethyl acetate. The CP fraction of ethyl acetate was concentrated and lyophilized. The extract of CP was stored in −70 °C until use.

### 2.3. Cell Culture and Cell Viability

HaCaT cells, a human keratinocyte cell line, were cultured in DMEM with 10% FBS and 1% P/S at 37 °C in 95% humidity using a 5% CO_2_ incubator. HMC-1 cells, a human mast cell line, were cultured in IMDM with 10% FBS and 1% P/S at 37 °C in 95% humidity with a 5% CO_2_ incubator. Subculture was conducted every 2-3 days. HaCaT cells were seeded in a 96-well cell culture plate with 1.5 × 10^4^ cells. After 24 h, CP was treated with concentrations of 0, 12.5, 25, 50, and 100 μg/mL for 24 h. HMC-1 cells were seeded in a 96-well plate with 1 × 10^5^ cells. After 24 h, CP was treated with concentrations of 0, 12.5, 25, 50, and 100 μg/mL for 24 h. Then, cell viability was measured using an MTS assay kit according to the manufacturer’s instructions. Absorbance was measured at 490 nm using a Versamax microplate reader (Molecular devices, San Jose, CA, USA).

### 2.4. Enzyme-Linked Immunosorbent Assay (ELISA)

HaCaT cells were seeded in a 6-well cell culture plate with 1 × 10^6^ cells. After stabilization for 24 h, CP (5, 10, and 20 μg/mL) was treated for 1 h, and the cells were treated with TNF-α/IFN-γ (each 10 ng/mL) for 24 h. After 24 h, the media were collected and centrifuged for 5 min at 4 °C and 13,200 rpm. HMC-1 cells were seeded in a 24-well cell culture plate with 3 × 10^5^ cells. After 24 h, CP (5, 10, and 20 μg/mL) was treated over 1 h, and the cells were treated with PMACI (PMA 25 nM/A23187 1 μM) for stimulation. After 6 h, all media were collected and decreased using a centrifuge for 5 min at 4 °C and 3000 rpm. The supernatants were used for cytokine analysis, and the cells were used for DNA expression analysis. Samples were stored at −70 °C. The cytokine levels of IL-6, IL-8, IL-1β, GM-CSF, MCP-1 and TNF-α were measured using ELISA kits (BD bio) according to the manufacturer’s instructions. Absorbance was measured using a Versamax microplate reader (Molecular devices, CA, USA).

### 2.5. Reverse Transcription Quantitative Polymerase Chain Reaction (RT-PCR)

RNA was extracted with Trizol according to the manufacturer’s instructions. cDNA was synthesized using SuperScript Ⅱ reverse transcriptase according to the manufacturer’s instructions. RT-PCR was performed using a KAPA Taq extra PCR kit on a Touch^TM^ thermal cycler (Bio-rad Lab, Hercules, CA, USA). The synthesized DNA was subjected to electrophoresis on 1.2% agarose gel containing N′,N′-dimethyl-N-[4-[(E)-(3-methyl-1,3-benzothiazol-2-ylidene)methyl]-1-phenylquinolin-1-ium-2-yl]-N-propylpropane-1,3-diamine (SYBR green I). The bands were captured using a NαBI^TM^. All bands were quantified using Image J software. The applied housekeeping gene was GAPDH. The used RT-PCR primer sequence is outlined in Table 1.

### 2.6. Western Blot

HaCaT cells were seeded in a 60 mm cell culture dish with 1 × 10^6^ cells. After 24 h, CP (20 μg/mL) was treated for 1 h; then, the TNF-α/IFN-γ (each 10 ng/mL) was treated for 5, 15, and 30 min (or 60 min). After that, the medium was removed in each well, and the cells were washed with DPBS 3 times. Cells were then lysed with a radioimmunoprecipitation assay buffer (RIPA buffer, 0.1% SDS, 150 mM NaCl, 50 mM Tris-cl, 1% nP-40, 0.5% Na-deoxycholate, PI_1_, PI_2_, PI_3_). Lysed cells were then collected with a cell scraper and centrifuged for 20 min at 4 °C and 13,200 rpm. Then, the supernatant was transferred to a new tube. The samples were stored at −70 °C until use. Nuclear protein was extracted using a nuclear extraction kit according to the manufacturer’s instructions. Samples were quantitatively analyzed using a bicinchoninic acid assay kit (BCA assay kit), and the same amount of protein was separated by sodium dodecyl sulphate polyacrylamide gel electrophoresis (SDS-PAGE) using 10% polyacrylamide gel and transferred to a nitrocellulose membrane. The membrane was blocked for 1 h with 5% skim milk and washed with tris-buffered saline including 0.05% Tween-20 (TBS-T). The membrane was then cultured at 4 ℃ overnight (O/N) with each primary antibody. Then, the membrane was cultured for 1 h at room temperature with a peroxidase-conjugated secondary antibody. Each expression was detected by X-ray film using an enhanced ECL solution. The relative protein levels were quantified using Image J software (ver. 1.53a, National Institutes of Health, Bethesda, MD, USA). The Western blot antibody data are presented in Table 2.

### 2.7. Animal Experiment

Six-week-old male Balb/c mice were purchased from Korean Animal Technology (KOATECH co, Seoul, Korea). All mice were bred in a controlled room (22 ± 2 °C temperature, 50 ± 10% humidity, 12 h light/dark cycle) and stabilized for 7 days. Mice were divided into 4 groups (*n* = 8/group): the normal group (Nor), the control group (only DNCB, Con), those treated with 1 mg/mL CP (with DNCB, CP_L), and those treated with 10 mg/mL CP (with DNCB, CP_H). This animal experiment was approved by the Kyung Hee Medical Center animal care and use committee (KHMC-IACUC-18-016).

### 2.8. ACD Model and Drug Treatment

**Figure 1:**
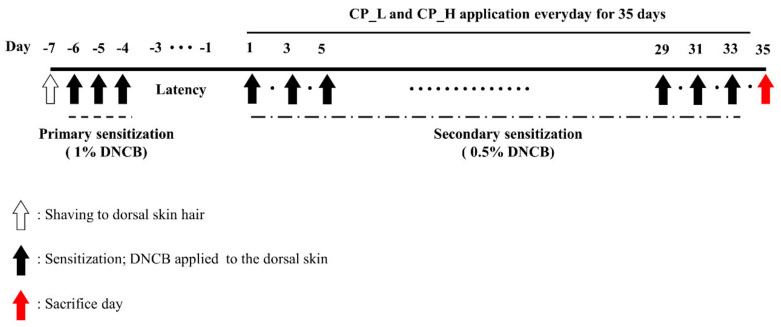
Schedule of animal experiments for 1-Chloro-2,4-dinitrobenzene (DNCB)-induced allergic contact dermatitis (ACD)-like lesions in the Balb/c mice model. Mice were divided into four groups (Nor, Con, CP-L, and CP-H groups). Each group was assigned eight mice. CP-L and CP-H were treated every day for 35 days.

All mice were anesthetized using an inhalation anesthetic by mixing 5% isoflurane with 100% oxygen. After anesthesia, 2–2.5% isoflurane was inhaled and maintained. The back of the mouse was shaved using a clipper under anesthesia. DNCB was dissolved in olive oil and acetone at a ratio of 3:1. To induce ACD-like skin lesions, DNCB was diluted in 1% and 0.5% and applied to the back skin of the mice. All mice except for the Nor group were primarily sensitized with 1% DNCB. Primary sensitization involved treatment of the dorsal skin with 200 μL of 1% DNCB over 3 days. After 5 days of primary sensitization, 200 μL of 0.5% DNCB was applied to the back skin for secondary sensitization 3 times a week for 5 weeks. CP was diluted in PBS/Olive oil (9:1). During secondary sensitization, 200 μL of CP_L and H was topically applied every day for 35 days. CP_L and H were topically applied to the dorsal skin 2 h after secondary sensitization. On the 36th day, all mice were exposed to 5% isoflurane to sacrifice them. It was confirmed that the heartbeat and breathing stopped after sustaining isoflurane exposure for about 5 min or more, after which 0.8–1 mL of blood was collected. The weights of the mice were 30–35 g at the time of sacrifice. The schedule of the animal experiment is shown in Figure 1.

### 2.9. Histological and Immunohistochemical (IHC) Staining

The skin tissue was fixed with Neutral Buffered Formalin (NBF) and washed with flowing water for O/N. Dehydration was carried out using ethanol, the cleaning response was studied using xylene, and the tissue was embedded in paraffin wax. Tissue blocks were sectioned with a 5 μm thickness using a rotary microtome (RM2125 RTS, Leica Biosystems, Wetzlar, Germany). The sectioned tissues were stained with hematoxylin and eosin (H&E) to measure the dermal and epidermal thickness and degree of eosinophil tissue infiltration. To observe the infiltration of mast cells, the tissue was stained with toluidine blue. The infiltration of CD4-positive T cells (CD4^+^ T cell) and CD8-positive T cells (CD8^+^ T cell) was confirmed by staining with IHC. Heat-induced epitope retrieval was performed in a 0.01 M sodium citrate buffer, pH 6.0, using a cooker (CPC-600, Cuisinart, CT, USA), and then cooled. Peroxidase activity was inhibited with 0.3% H_2_O_2_ (*w*/*v*) in methanol at room temperature for 30 min. The tissues were washed twice with TBS and blocked with 10% normal serum in TBS for 30 min. The tissues were then incubated with CD4^+^ or CD8^+^ antibodies at 4 °C for 24 h. The color reaction of CD4^+^ T cells or CD8^+^ T cells was examined using a Polink-2 Plus AP rabbit kit (D70-18, GBI Labs, WA, USA) according to the manufacturer’s instructions. The background was stained with hematoxylin. Stained tissue was observed by a light microscope (BX51, Olympus, Tokyo, Japan) at ×400 magnification. The data on antibodies stained using IHC are provided in Table 1.

### 2.10. Clinical Skin Severity Score

The effect of CP in DNCB-induced ACD-like skin lesion mice was estimated by the changes in severity of skin lesions (modified SCORAD, Scoring atopic dermatitis). Severity of ACD-like skin lesions was evaluated on the day of the sacrifice as follows. The dorsal lesions were evaluated for 6 symptoms: redness, swelling, oozing (or crusting), scratching marks, skin thickening, and dryness. Each symptom was graded from 1 to 3 (none, 0; mild, 1; moderate, 2; serve, 3). The score was defined as the sum of the individual scores. The SCORAD evaluation was performed after group blinding.

### 2.11. Statistical Analysis

All experiments were repeated at least three times independently. Data are presented as the mean ± standard error of the mean (SEM). Statistical analysis was performed using Graphpad PRISM software (Ver 7.00, Graphpad software Inc., San Jose, CA, USA). A *t*-test or one-way analysis of variance (ANOVA) was used to evaluate the treatment effect, followed by Dunnett’s multiple comparison test. *p* values of *p* < 0.05 and *p* < 0.01 were considered significant.

## 3. Results

### 3.1. Cell Viability of CP and the Effects of CP for Pro-Inflammatory Cytokines in HaCaT Cells

**Figure 2:**
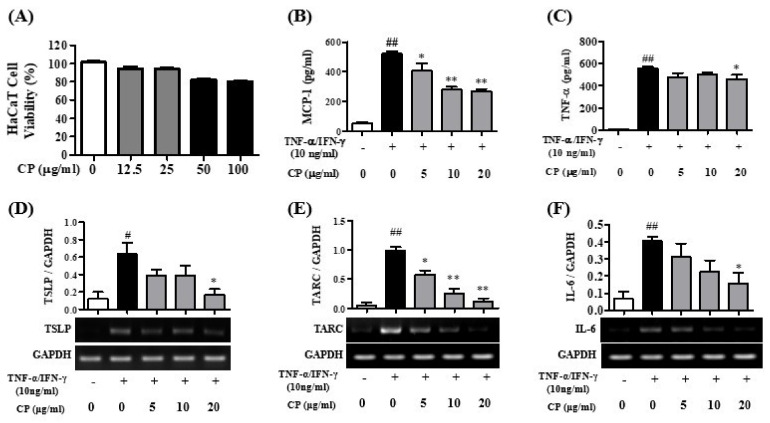
Effect of *Cone of Pinus densiflora* (CP) on TNF-α/IFN-γ-stimulated HaCaT cells. (**A**) Viability of CP in HaCaT cell by MTS assay. HaCaT cells were treated with different concentrations of CP (0, 12.5, 25, 50 and 100 μg/mL) for 24 h. (**B**,**C**) MCP-1, TNF-α expression in TNF-α/IFN-γ-stimulated HaCaT cells. The cells were treated with different concentrations of CP (5, 10 and 20 μg/mL) for 1 h and then treated with TNF-α/IFN-γ (10 ng/mL) for 24 h. Absorbance was measured using a microplate reader at 490 nm. (**D**–**F**) mRNA levels of inflammatory cytokine in TNF-α/IFN-γ-stimulated HaCaT cells. The cells were treated with different concentrations of CP (5, 10 and 20 μg/mL) for 1 h and then treated with TNF-α/IFN-γ (10 ng/mL) for 3 h (TSLP) of 24 h (TARC and IL-6). The mRNA expression level of TSLP, TARC and IL-6 was quantitative, analyzed by comparing it with GAPDH in the Image J Program. All data represent the means ± SEM (^#^ *p* < 0.05 and ^##^ *p* < 0.01 vs. TNF-α/IFN-γ non-treat group. * *p* < 0.05 and ** *p* < 0.01 vs. only TNF-α/IFN-γ-treat group).

To confirm the cytotoxicity of CP in HaCaT cells, CP was treated for 24 h at 0, 12.5, 25, 50, and 100 μg/mL in HaCaT cells. There was no cytotoxicity at 12.5 and 25 μg/mL, but toxicity began to appear at 50 μg/mL in HaCaT cells (Figure 2A). Therefore, the HaCaT cell experiment was conducted at concentrations of 5, 10, and 20 μg/mL, which are concentrations that did not show toxicity.

The inhibition of pro-inflammatory cytokines for CP in TNF-α/IFN-γ-stimulated HaCaT cells was analyzed using an ELISA kit. The expression levels of the pro-inflammatory cytokines MCP-1 and TNF-α were significantly increased in the Con group compared to the Nor group in the HaCaT cells (Figure 2B,C). When the HaCaT cells were treated with CP, the MCP-1 expression level significantly decreased at concentrations of 5, 10, and 20 μg/mL of CP compared with the Con group (Figure 2B), and the TNF-α expression level was significantly inhibited at a concentration 20 μg/mL CP compared with the Con group (Figure 2C) in HaCaT cells.

Expression of the pro-inflammatory cytokines TSLP, TARC and IL-6 mRNA was confirmed in HaCaT cells. RT-PCR was used to confirm whether CP inhibited expression of the pro-inflammatory cytokines TSLP, TARC and IL-6 mRNA in HaCaT cells (Figure 2). TSLP, TARC and IL-6 mRNA levels significantly increased in the Con group compared with the Nor group in the HaCaT cells. CP significantly decreased TARC mRNA at 5, 10, and 20 μg/mL concentrations compared with the Con group (Figure 2E). Moreover, CP markedly decreased TSLP and IL-6 mRNA levels at 20 μg/mL compared with the Con group (Figure 2D,F).

### 3.2. Cell Viability of CP and the Effect of CP on Pro-Inflammatory Cytokines in HMC-1 Cells

**Figure 3:**
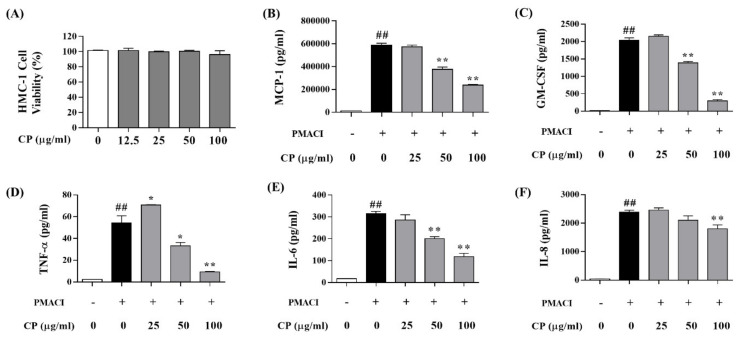
Effect of CP on phorbol-12-myristate-13-acetate plus A23187 (PMACI)-stimulated HMC-1 cells. (**A**) Viability of CP in HMC-1 cell by MTS assay. HMC-1 cells were treated with different concentrations of CP (0, 12.5, 25, 50 and 100 μg/mL) for 24 h. (**B**–**F**) Inflammatory cytokine expression in PMACI-stimulated HMC-1 cells. The cells were treated with the different concentrations of CP (25, 50 and 100 μg/mL) for 1 h and then treated with PMACI for 7 h. Absorbance was measured using a microplate reader at 490 nm. All data represent the means ± SEM (^##^ *p* < 0.01 vs. TNF-α/IFN-γ non-treat group. * *p* < 0.05 and **p < 0.01 vs. only TNF-α/IFN-γ-treat group).

The cytotoxicity of CP was measured in HMC-1 cells. CP was administered at 0, 12.5, 25, 50, and 100 μg/mL concentrations for 24 h in HMC-1 cells. HMC-1 cells showed no cytotoxicity at all concentrations of CP (Figure 3A). Therefore, the HMC-1 cells experiment was conducted at concentrations of 12.5, 25, 50, and 100 μg/mL.

The inhibition of pro-inflammatory cytokines for CP in PMACI (PMA 25 nM/A23187 1 μM)-stimulated HMC-1 cells was analyzed using an ELISA kit. MCP-1 (Figure 3B), GM-CSF (Figure 3C), TNF-α (Figure 3D), IL-6 (Figure 3E), and IL-8 (Figure 3F) expression levels significantly increased in the Con group compared with the Nor group in HMC-1 cells. When HMC-1 cells were treated with CP, the MCP-1 expression level significantly decreased at concentrations of 50 and 100 μg/mL of CP compared with the Con group (Figure 3B), and the GM-CSF expression level significantly inhibited at concentrations of 50 and 100 μg/mL of CP compared with the Con group in HMC-1 cells (Figure 3C). TNF-α expression level significantly decreased at a concentration 50 and 100 μg/mL of CP compared with the Con group in HMC-1 cells (Figure 3D). IL-6 expression level significantly decreased at concentrations of 50 and 100 μg/mL of CP compared with the Con group (Figure 3E). IL-8 expression level markedly decreased at a concentration of 100 μg/mL of CP compared with the Con group (Figure 3F).

### 3.3. Effect of CP on the MAPKs and NF-κB/IκBα Signaling Pathway in TNF-α/IFN-γ-Stimulated HaCaT Cells

**Figure 4:**
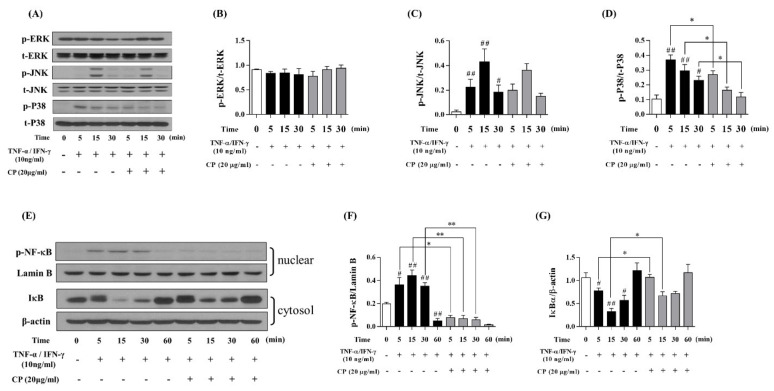
Effect of CP on MAPK signaling pathway in TNF-α/IFN-γ-stimulated HaCaT cells. (**A**–**D**) HaCaT cells were treated with CP (20 μg/mL) for 1 h and then stimulated with TNF-α/IFN-γ for 5, 15 and 30 min. The phosphorylation of MAPKs was analyzed by western blot analysis. Phosphorylations of MAPKs (ERK, JNK, P38) were normalized to total MAPKs. (**E**–**G**) HaCaT cells were treated with CP (20 μg/mL) for 1 h and then stimulated with TNF-α/IFN-γ for 5, 15, 30 and 60 min. The phosphorylation of the NF-κB/IκBα signaling pathway was analyzed using western blot analysis. p-NF-κB was normalized to Lamin B, and IκBα was normalized to β-actin. All data represent the means ± SEM (^#^ *p* < 0.05 and ^##^ *p* < 0.01 vs. TNF-α/IFN-γ non-treat group. * *p* < 0.05 and ** *p* < 0.01 vs. only TNF-α/IFN-γ-treat group).

Western blot was performed to confirm whether CP suppresses the MAPKs signaling pathway. The phosphorylation of ERK, JNK, and P38 increased in the Con group compared with the Nor group (Figure 4A). CP was not affected by the phosphorylation of ERK and JNK (Figure 4B,C), but CP inhibited the phosphorylation of P38 at 5, 15, and 30 min (Figure 4D).

After confirming that CP suppresses the expression of P38, the effect of CP on the NF-κB/IκBα signaling pathway downstream from MAPKs signaling was analyzed. Western blot was performed to confirm whether CP suppresses the NF-κB/IκBα signaling pathway. The phosphorylation of NF-κB increased in the Con group compared with the Nor group, and IκBα decreased in the Con group compared with the Nor group (Figure 4E). However, CP significantly decreased NF-κB translocation compared with the Con group at 5, 15, and 30 min (Figure 4F). Moreover, CP significantly increased IκBα compared with the Con group at 5 and 15 min (Figure 4G).

### 3.4. Effect of CP on DNCB-Induced ACD-Like Skin Lesion in the Balb/c Mouse Model

**Figure 5:**
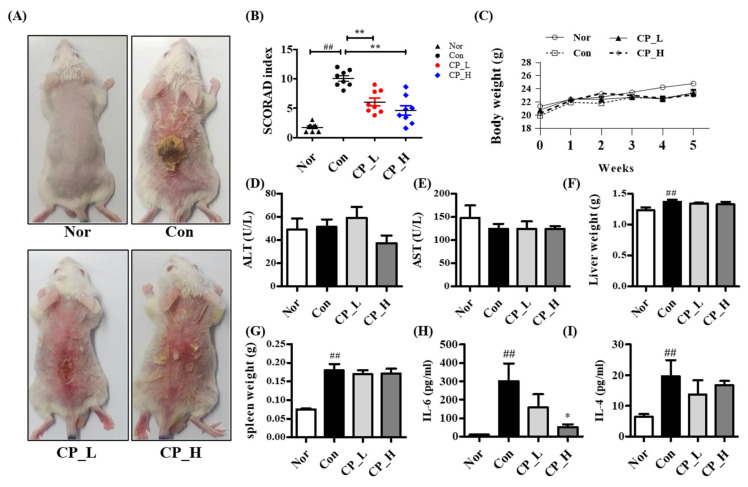
Effect of CP in DNCB-induced ACD-like skin lesion. (**A**) CP was treated in DNCB-induced an ACD-like skin lesion mice model for 5 weeks. (**B**) The SCORAD index of ACD-like skin lesion mice (*n* = 8 per group) was evaluated. (**C**) Body weight was measured once a week. (**D**–**F**) Level of alanine aminotransferase (ALT) and aspartate aminotransferase (AST) and liver weight were measured to examine for liver toxicity and function. (**G**) Spleen weight was measured to confirm the systemic immune response. (**H**,**I**) Levels of IL-6 and IL-4 in tissue lysate were measured by ELISA. All data represent the means ± SEM (^##^ *p* < 0.01 vs. Nor group. * *p* < 0.05 and ** *p* < 0.01 vs. Con group).

To confirm the effect of CP in the ACD-like skin lesion model, after inducing an ACD-like skin lesion with DNCB, CP was applied to the dorsal skin for 5 weeks. ACD lesions such as redness, swelling, oozing, scratching marks, skin thickening, and dryness occurred in the Con group. The SCORAD index revealed significantly improved symptoms of redness, swelling, oozing, scratching marks, skin thickening, and dryness in the CP-L and CL-H groups. There were more that significantly decreased in CL-H group than CL-L group (Figure 5A,B). The all mice’s weights were not unusual (Figure 5C). The levels of alanine aminotransferase (ALT) and aspartate aminotransferase (AST) and liver weight were measured to confirm liver damage and toxicity. Spleen weights were measured to investigate the systemic immune response. As shown Figure 5D–G, CP application did not affect the liver and spleen. Additionally, we measured the levels of IL-4 and IL-6 in tissue to confirm the effect of CP in the expression of inflammatory cytokine in skin lesion. As shown Figure 5H,I, CP-H significantly decreased expression of IL-6 but did not affect the expression of IL-4.

### 3.5. Effect of CP on the Epidermal or Dermal Thickness and Infiltration of Eosinophils, Mast Cells, CD4^+^ and CD8^+^ T Cells 

**Figure 6:**
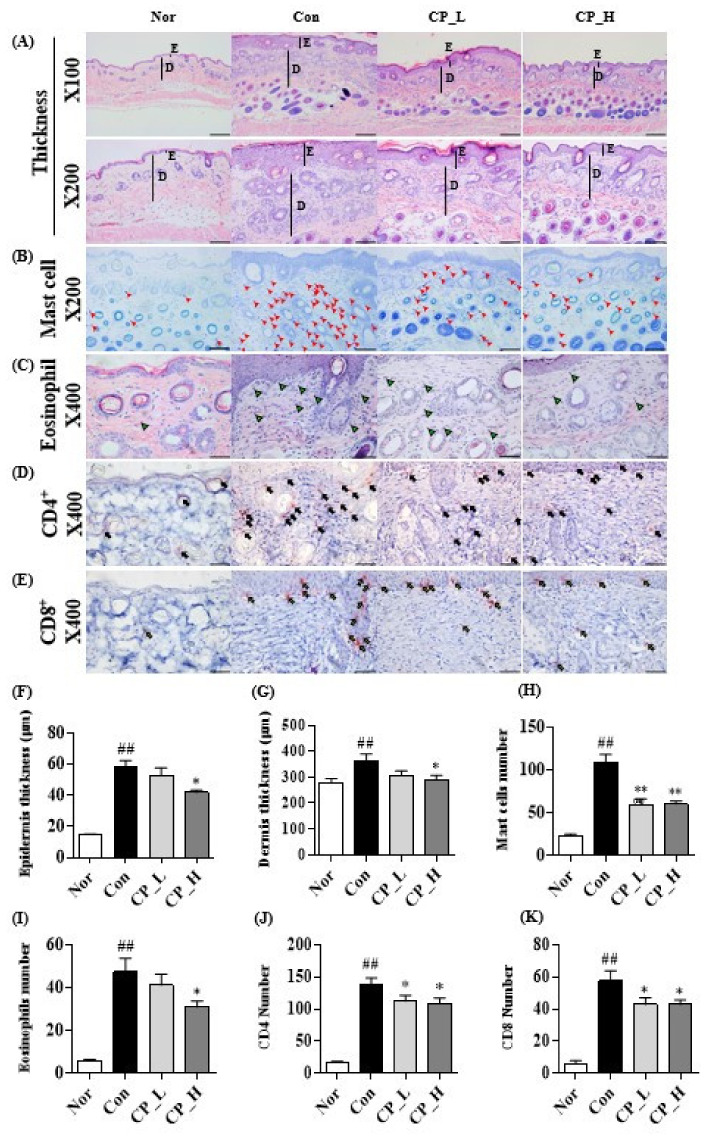
Effect of CP on immunohistochemical (IHC) and histological staining of DNCB-induced ACD-like skin lesions in mice. (**A**,**E**–**G**) Epidermal and dermal thickness were assessed via H&E staining (×100, scale bar 200 μm; ×200, scale bar 100 μm). (**B**,**H**) Mast cells (red arrow heads) were measured by toluidine blue staining (×200, scale bar, 100 μm). (**C**,**I**) Eosinophils (green arrow heads) were counted after hematoxylin and eosin staining (×400, scale bar 50 μm). (**D**,**J**) Infiltration of CD4^+^ T cells (black arrows) was examined by IHC staining of skin tissue sections (×400, scale bar 50 μm). (**E**,**K**) Infiltration of CD8^+^ T cells (yellow arrows) was examined by IHC staining of the skin tissue sections (×400, scale bar 50 μm). All data represent the means ± SEM (^##^ *p* < 0.01 vs. Nor group. * *p* < 0.05 and ** *p* < 0.01 vs. Con group).

H&E and toluidine blue staining were performed to evaluate the efficacy of CP in skin lesions. Epidermal and dermal thickness significantly increased in the Con group compared with the Nor group (Figure 6A). However, CP_L or CP_H significantly decreased epidermal and dermal thickness compared with the Con group (Figure 6F,G). The infiltration of mast cell was confirmed in the toluidine-blue-stained skin tissue. The tissue infiltration of mast cells markedly increased in the Con group compared with the Nor group (Figure 6B). Both CP_L and CP_H significantly suppressed the infiltration of mast cells into the skin lesions (Figure 6H). H&E staining was performed to observe the infiltration of eosinophils into the skin tissue. The infiltration of eosinophil significantly increased in the Con group compared with the Nor group (Figure 6C), but CP_L or CP_H significantly suppressed the infiltration of eosinophils into the skin lesions (Figure 6I).

IHC staining was performed to verify the effects of CP on the infiltration of CD4^+^ T and CD8^+^ T cells into the skin lesions. Skin infiltration of CD4^+^ T cells markedly increased in the Con group compared with the Nor group (Figure 6D), but both CP_L and CP_H significantly reduced the infiltration of CD4^+^ T cells into the skin lesions. (Figure 6J). Skin infiltration of the CD8^+^ T cells markedly increased in the Con group compared with the Nor group (Figure 6E), but both CP_L and CP_H significantly reduced the infiltration of CD8^+^ T cells into the skin lesions (Figure 6K).

## 4. Discussion

The purpose of this study was to evaluate the effects of CP on TNF-α/IFN-γ-stimulated HaCaT cells, PMACI-stimulated HMC-1 cells, and DNCB-induced ACD-like skin lesions. This study confirmed the anti-inflammatory effect through the intracellular inflammatory response mechanism in HaCaT cells stimulated with TNF-α/IFN-γ and the HMC-1 cells model stimulated with PMACI, which is mainly used in allergic contact dermatitis research, as well as DNCB-induced ACD-like skin lesions. The anti-inflammatory effect of CP on the pathological and histological symptoms of ACD-like skin lesions in a Balb/c mouse model was also verified.

In ACD, TSLP secreted by epidermal keratinocyte is a trigger that induces dendric-cell-mediated allergic inflammation. Dendritic cells activated by TSLP enhance Th2-mediated inflammation by producing chemokines such as TARC [14]. The actions of TARC and MCP-1 play an important role in the inflammatory responses of allergic contact dermatitis [15,16]. These chemokines serve to collect other inflammatory cells at the site of the inflammatory reaction and play a role in sustaining inflammation. GM-CSF is mainly secreted from keratinocyte and mast cells and promotes the stimulation of LCs to eosinophils, neutrophils, and basophils, causing chronic allergic contact dermatitis [22]. IL-6 plays an important role in inflammation and immunity by inducing the maturation of Th2 cells, the inhibition of various macrophage functions, and the activation and proliferation of eosinophils and mast cells [21]. IL-8 acts extensively on various types of cells, including neutrophils, monocytes, endothelial cells, and fibroblasts, and for this reason, it plays an important role in inflammatory diseases such as chronic inflammation [23]. Therefore, the response of pro-inflammatory cytokines in TNF-α/IFN-γ-stimulated HaCaT cells and PMACI-stimulated HMC-1 cells was confirmed.

As a result of the experiment, CP treatment significantly decreased the expression of TARC, MCP-1, IL-6, and TNF-α in stimulated HaCaT cells and markedly decreased the expression of MCP-1, GM-CSF, TNF-α, IL-6, and IL-8 in HMC-1 cells. These results indicate that CP exerts anti-inflammatory effects by inhibiting inflammatory chemokines and cytokines secreted from stimulated HaCaT cells and stimulated HMC-1 cells. Based on the experimental results, to determine through which mechanism the anti-inflammatory effect of CP is achieved, the MAPKs signaling pathway, which is the most well-known inflammatory reaction mechanism, and the NF-κB/IκBα signaling pathway, which is well-known as being downstream of MAPKs, were identified.

Inflammatory cytokines are mainly expressed in the MAPKs signaling pathway and the NF-κB/IκBα signaling pathway, a downstream pathway [28]. P38, part of the MAPKs family, is primarily involved in the inflammatory response [30]. P38 phosphorylated by external stimuli influences downstream signaling [30,31]. In the NF-κB/IκBα signaling pathway, which is well known as a signaling pathway downstream of MAPK, the inactive NF-κB complex, which exists in a normal state, is phosphorylated by P38, causing NF-κB and IκBα to separate from each other, and the phosphorylated NF-κB is then translocated to the nucleus, and IκBα is degraded [32,33]. The nuclear translocation of NF-κB is involved in the expression of numerous genes involved in various immune system and inflammatory responses causing ACD [33]. Thus, the effect of CP was confirmed for the MAPKs signaling pathway and the NF-κB/IκBα signaling pathway in the TNF-α/IFN-γ-stimulated HaCaT cells.

In the study results, CP inhibited the phosphorylation of P38 in the activated MAPK family of stimulated HaCaT cells, inhibited the nuclear translocation of NF-κB, and inhibited degradation due to the phosphorylation of IκBα. These results suggest that CP inhibits the effects downstream by suppressing the phosphorylation of P38 in the major intracellular inflammatory response mechanisms; it has a positive effect by inhibiting the phosphorylation of IκBα in the inactive NF-κB complex, thus preventing stimulation of upstream. It was also confirmed that inhibiting the phosphorylation of NF-κB inhibits the secretion of inflammatory cytokines by suppressing the nuclear translocation of NF-κB.

This study confirmed the intracellular inflammatory reaction caused by the effect of CP on the expression and secretion of inflammatory cytokine. Based on this study, animal experiments were conducted to confirm the effect of CP on ACD-induced skin lesion. The main symptoms of ACD-lesions are increased thickness of the epidermal and dermal layers [46,47]. ACD causes the infiltration of various inflammatory cells, hyperkeratosis of keratinocytes from inflammatory reactions, and induces the epidermis and dermis to become thick and hard, leading to rashes, eczema, persistent scratching, and erythema [48]. Increased eosinophils and mast cells in ACD lesions secrete various inflammatory cytokines and chemokines in the tissue to induce inflammation and other inflammation-inducing cells, thereby further exacerbating the lesion site in the skin [25,49]. Thus, the DNCB-induced ACD-like skin lesions model was used to confirm the effect of CP on pathological symptoms, organ toxicity, and histological symptoms. 

Toxicity of CP was tested by observing conditions such as skin, physical activity, behavior patterns, diarrhea, convulsions, tremors, and lethargy according to the recommendations of the Organization for Economic Cooperation and Development (OECD) [50]. AST and ALT data and liver and spleen weights were measured after mouse sacrifice to prove again that there was no toxicity at the corresponding concentration. The pathological symptoms of ACD were visually confirmed by the SCORAD index. As a result of the study, CP alleviated the pathological symptoms of allergic contact dermatitis-like skin lesions. These results showed that CP has a positive effect on ACD-like skin lesions, and a histological experiment was conducted to determine whether the pathological symptoms of ACD-like lesions were alleviated and by what kind of action.

CP was confirmed to decrease the thickness of the epidermis and dermis through histological experiments, thus confirming the reason why the thickness of the epidermis and dermis was decreased by CP. Moreover, the importance of the infiltration of inflammatory cells in the inflammatory response of the tissue was also confirmed. H&E was performed to confirm the infiltration of eosinophil and confirmed that eosinophil infiltration was decreased by CP. Moreover, toluidine blue staining was performed to confirm that the infiltration of the mast cells was decreased by CP.

CD4^+^ T cells and CD8^+^ T cells refer to lymphocytes with a marker called CD4 or CD8 on the cell surface [51]. These cells perceive the allergens presented by antigen-presenting cells (APCs) and activate T-cells via the interaction of the major histocompatibility complex class (MHC) and T-cell receptor (TCR) [52]. The activated T-cells differentiate into several types of T-helper cells, secrete various inflammatory cytokines, and trigger an inflammatory reaction [53]. As a result of the experiment, it was also confirmed that CP decreases the infiltration of CD4^+^ T and CD8^+^ T cells in the inflammatory response of ACD.

In summary, CP inhibited activation of the MAPK family P38 and the NF-κB/IκBα signaling pathway, thereby inhibiting the expression of TARC, MCP-1, TNF-α, and IL-6 in HaCaT cells and the expression of MCP-1, GM-CSF, TNF-α, IL-6, and IL-8 in HMC-1 cells. CP reduced the pathological symptoms of ACD and reduced histological symptoms such as epidermal and dermal thickness, the infiltration of eosinophils, mast cells, CD4^+^ T cells, and CD8^+^ T cells in vivo. These results suggest that CP may be an effective alternative medicine for ACD-like lesions.

To conclude, in the ACD inflammatory response, CP inhibits the differentiation process of Th cells via CD4^+^ T cells and CD8^+^ T cells, thereby reducing the secretion of inflammatory cytokines and inhibiting eosinophil and mast cell infiltration to alleviate the inflammatory response. This response reduces the thickness of the epidermis and dermis.

In many studies, the composition of *Cone of Pinus densiflora* has been studied. The terpene group, known as the main component of pinecones, includes, e.g., alpha-pinene, beta-pinene, and abietic acid. Alpha-pinene and beta-pinene contribute to the anti-inflammatory reaction, and abietic acid has inhibited the translocation of NF-κB. As a result of the studies, the anti-inflammatory effect of CP is shown by the efficacy of the terpene-based components known to be contained in drops. However, since the component of CP used in the experiment was not clearly identified, it appears that additional experiments are needed. HPLC should be performed to clarify the CP components, and it is necessary to compare the efficacy of the components identified by performing HPLC with a CP acetate extract. In later studies, we will conduct a comparative experiment of the HPLC and CP extracts and the components identified in the HPLC.

## Figures and Tables

**Table 1:** Primer Sequence and PCR conditions.

Gene Name	Orientation	Primer Sequence	Anneling Tm (°C)	Cycle	Reference
TSLP	Forward Reverse	5′-TCC TCT GAA GAC CTG ACC-3′ 5′-TCT CCT TTC TCC CTA ATC CTC-3′	59.5 °C	40	kim et al. [45]
TARC	Forward Reverse	5′-ACT GCT CCA GGG ATG CCA TCG TTT TT-3′ 5′-ACA AGG GGA TGG GAT CTC CCT CAC TG-3′	57.5 °C	44	NM_002987.3
IL-6	Forward Reverse	5′-GAT GGC TGA AAA AGA TGG ATG C-3′ 5′-TGG TTG GGT CAG GGG TGG TT-3′	59 °C	45	NM_000600.4
GAPDH	Forward Reverse	5′-CGT CTA GAA AAA CCT GCC AA-3′ 5′-TGA AGT CAA AGG AGA CCA CC-3′	50 °C	30	NM_001256799.3

Abbreviations: TSLP, Thymic stromal lymphopoientin; TARC/CCL17, Thymus and activation-regulated chemokine; IL-6, Interleukin 6; GAPDH, glyceraldehyde-3phosphate dehydrogenase.

**Table 2:** Western blot and immunohistochemical primary anti-body and secondary anti-body conditions.

Primary Antibody	Primary Antibody Dilution	System Used	Size
Phospho-ERK	1:1000	Western blot	42, 44 kDa
Phospho-JNK	1:1000	Western blot	46, 54 kDa
Phospho-P38	1:1000	Western blot	43 kDa
ERK	1:1000	Western blot	42, 44 kDa
JNK	1:1000	Western blot	46, 54 kDa
P38	1:1000	Western blot	43 kDa
Phospho-NF-κB	1:1000	Western blot	65 kDa
IκBα	1:1000	Western blot	39 kDa
Lamin B	1:1000	Western blot	67 kDa
β-actin	1:500	Western blot	43 kDa
CD4^+^	1:200	Immunohistochemistry	-
CD8^+^	1:200	Immunohistochemistry	-
Goat-anti-Rabbit IgE	1:10,000	Western blot	-
Goat-anti-mouse-IgE	1:10,000	Western blot	-

Abbreviations: Phospho, Phosphorylation; ERK, extracellular signal-regulated kinases; JNK, c-Jun N-terminal kinase; NF-κB, nuclear factor-kappa B; IκBα, nuclear factor of kappa light polypeptide gene enhancer in B-cells inhibitor alpha; cluster of differentiation 4, CD4; cluster of differentiation 8, CD8; IgE, Immunoglobulin E.

## Data Availability

The data presented in this study are available on request from the corresponding author.
